# Addressing the Comorbidity Between Epilepsy and Psychiatric Disorders

**DOI:** 10.1192/j.eurpsy.2024.984

**Published:** 2024-08-27

**Authors:** A. H. I. Abu Shehab, T. Simona, U. Ion, D. C. Voinescu, C.-F. Buciu, A. Ciubară

**Affiliations:** ^1^Psychiatry Department, “Elisabeta Doamna” Psychiatric Hospital, Galati; ^2^Psychiatry Department, Carol Davila University of Medicine and Pharmacy, Bucharest; ^3^Psychiatry Department, University of Medicine and Pharmacy of Craiova, Craiova; ^4^Rheumatology department, “Dunărea de Jos” University - Faculty of Medicine and Pharmacy, Galati; ^5^public health and management, University of Medicine, Pharmacy, Science and Technology of Târgu Mureș, Târgu Mureș; ^6^Psychiatry Department, “Dunărea de Jos” University - Faculty of Medicine and Pharmacy, Galati, Romania

## Abstract

**Introduction:**

The intricate and multifaceted nature of the link between epilepsy and psychiatric diseases is evident. Patients diagnosed with epilepsy frequently exhibit concurrent psychiatric illnesses, including but not limited to depression, anxiety, psychosis, and attention-deficit disorders. Gaining a comprehensive understanding of the fundamental mechanisms and implementing efficacious ways to effectively address this co-occurring medical condition is crucial in order to achieve the most advantageous results for patients.

**Objectives:**

The objective of this study is to examine the frequency, neurobiological bases, and consequences for treatment of psychiatric comorbidities in patients diagnosed with epilepsy. The study aims to offer a thorough understanding of the subject and promote interdisciplinary collaboration.

**Methods:**

A systematic review of literature was conducted, focusing on clinical studies, neuroimaging findings, and neurochemical changes in patients with both epilepsy and psychiatric disorders. Additionally, best-practice recommendations for the clinical management of this patient population were identified.

**Results:**

The results suggest that the coexistence of epilepsy and psychiatric diseases may be affected by neuroinflammation, abnormalities in neurotransmitters, and shared genetic factors. In addition, the implementation of integrated therapy techniques that include both neurological and psychological components has demonstrated encouraging findings in enhancing patient outcomes.

**Conclusions:**

The identification and proficient management of psychiatric comorbidities in individuals with epilepsy are of utmost significance. The establishment of interdisciplinary collaboration between neurologists and psychiatrists, supported by continuous research, is necessary in order to provide comprehensive treatment and enhance the overall well-being of individuals affected by these conditions.

**Disclosure of Interest:**

None Declared

